# Identification of novel extracellular putative chitinase and hydrolase from *Geomyces* sp. B10I with the biodegradation activity towards polyesters

**DOI:** 10.1186/s13568-022-01352-7

**Published:** 2022-02-05

**Authors:** Aneta K. Urbanek, Miguel Arroyo, Isabel de la Mata, Aleksandra M. Mirończuk

**Affiliations:** 1 Department of Biotechnology and Food Microbiology, Faculty of Biotechnology and Food Science, Wroclaw University of Environmental and Life Sciences, Chełmońskiego 37, 51‑630 Wrocław, Poland; 2 Department of Biochemistry and Molecular Biology, Faculty of Biology, Universidad Complutense de Madrid, C. de José Antonio Novais, 12, 28040 Madrid, Spain

**Keywords:** *Geomyces* sp. B10I, Polyesters, Chitinase, Hydrolase, Cold-adapted microorganisms

## Abstract

Cold-adapted filamentous fungal strain *Geomyces* sp. B10I has been reported to decompose polyesters such as poly(e-caprolactone) (PCL), poly(butylene succinate) (PBS) and poly(butylene succinate-co-butylene adipate) (PBSA). Here, we identified the enzymes of *Geomyces* sp. B10I, which appear to be responsible for its biodegradation activity. We compared their amino acid sequences with sequences of well-studied fungal enzymes. Partial purification of an extracellular mixture of the two enzymes, named hydrGB10I and chitGB10I, using ammonium sulfate precipitation and ionic exchange chromatography gave 14.16-fold purity. The amino acid sequence of the proteins obtained from the MALDI-TOF analysis determined the molecular mass of 77.2 kDa and 46.5 kDa, respectively. Conserved domain homology analysis revealed that both proteins belong to the class of hydrolases; hydrGB10I belongs to the glycosyl hydrolase 81 superfamily, while chitGB10I contains the domain of the glycosyl hydrolase 18 superfamily. Phylogenetic analysis suggests a distinct nature of the hydrGB10I and chitGB10I of *Geomyces* sp. B10I when compared with other fungal polyester-degrading enzymes described to date.

**Supplementary Information:**

The online version contains supplementary material available at 10.1186/s13568-022-01352-7.

## Introduction

Conventional plastics are an integral part of our daily activities and make a significant contribution to various industries. Unfortunately, the extensive use of plastic causes severe environmental problems. One solution is to develop and commercialize biodegradable plastics such as polybutylene succinate (PBS), polybutylene succinate-co-adipate (PBSA), polycaprolactone (PCL), polylactic acid (PLA) or polyhydroxybutyrate (PHB), which are decisively more environment-friendly than fossil-based plastics (Nawaz et al. 2015; Jung et al. 2018). Due to their sensitivity to the enzymatic attack of microorganisms, the physical and chemical structure of the material and the decomposition conditions, these polyesters can naturally degrade in the environment (Emadian et al. 2017). Many microorganisms and microbial enzymes have been isolated and described as effective biocatalysts for the degradation of biodegradable plastics. A particularly large number of hydrolases across many species of microorganisms have been biochemically and structurally characterized (Lee and Huang 2013). Hydrolases represent an important class of enzymes that can increase the rate of degradation of biomass to chemical precursors and can also be effective in removing microplastics from water sources, which is a rapidly growing environmental threat (Johnson et al. 2021). The most frequently mentioned hydrolases revealing activity against polyester-based plastics are lipases, cutinases, esterases, proteases and depolymerases (Liu et al. 2019; Urbanek et al. 2020). It should be emphasized that among many known enzymes of microbial origin, fungal enzymes with biodegradation activity towards plastic are still little known, and most of them have been reported from *Penicillium* and *Aspergillus* species (Kim and Rhee 2003). For instance, *Penicillium funiculosum* and *Penicillium expansum* have been reported to produce PHB depolymerase (Brucato and Wong 1991; Gowda and Shivakumar 2015). *Aspergillus oryzae* RIB40 was able to degrade PBS and PBSA due to secretion of cutinase (Maeda et al. 2005; Liu et al. 2009), whereas PHB depolymerase from *Aspergillus fumigatus* 76 T-3 could decompose PHB and PES (polyethersulfone). Many other filamentous fungi, belonging to *Fusarium* (Abe et al. 2010; Sameshima-Yamashita et al. 2016; Mao et al. 2005; Urbanek et al. 2021), *Clonostachys*, *Trichoderma* (Urbanek et al. 2017), *Geomyces*, *Sclerotinia*, *Mortierella* (Urbanek et al. 2021), *Alternaria* (Abdel-Motaal et al. 2020) or other have been reported as being able to degrade bioplastic without simultaneous enzymatic characterization. It proves that the enzymatic potential of fungi has not been sufficiently discovered yet.

In our previous study, we isolated the cold-adapted fungus *Geomyces* sp. B10I from Antarctic soil samples collected in the vicinity of Arctowski Polish Antarctic Station on King George Island, which has been reported to exhibit biodegradation activity towards the polyesters PBSA, PBS and PCL (Urbanek et al. 2021). Species in this filamentous genus tend to be keratinophilic and psychrophilic. *Geomyces* are found in a variety of ecosystems and are often the most common group of fungi found in cold environments such as Arctic permafrost, Antarctic soil, and even glacier bank soils at over 3000 m above sea level (Hayes 2012). Since *Geomyces* sp. is a cold-adapted fungus, likely its enzymes can be active at low temperatures. The use of cold-adapted enzymes may reduce the need of heating, increase the quality, sustainability and cost-effectiveness of technological processes (Santiago et al. 2016). Moreover, these excellent biocatalysts may play a role in the biodegradation of plastic.

Here, we partially purified and characterized two novel enzymes produced by *Geomyces* sp. B10I. Based on the basic local alignment search tool (BLAST) (Altschul et al. 1990), both proteins were found to belong to the hydrolase class, and more specifically to the hydrolase and chitinase families. As hydrolases are common enzymes with biodegradable activity, both enzymes are perceived to degrade polyesters and have been named hydrGB10I and chitGB10I, respectively.

## Materials and methods

### Fungal strain and culture media

The cold-adapted strain of filamentous fungi *Geomyces* sp. B10I with PBSA/PBS/PCL-degrading activity deposited in Collection of Industrial Microorganisms (IAFB) (Warsaw, Poland) under number KPP 3680 was used in this study. For the enzyme production 2xYT medium composed of tryptone 1.6%, yeast extract 1.0%, NaCl 0.5% and Tween 80 0.1% was used. Ten flasks containing 100 mL of 2xYT liquid medium were inoculated with 2–3 loops (20–30 µg) of mycelium taken from the plate culture and incubated at 21 °C, 140 rpm for 72–96 h. Minimal mineral (MM) medium containing 0.1% emulsified polyesters (PBSA, PBS or PCL) was prepared for the examination of BP-degrading enzymatic activity as previously described (Urbanek et al. 2021). Cell-free broth, fractions after stepwise precipitation with ammonium sulfate and fractions collected after IEC were preserved at 4 or − 20 °C.

### Materials

Polybutylene succinate (PBS) and polybutylene succinate-co-adipate (PBSA) under the trade names Bionolle 1020MD and Bionolle 3020MD, respectively, were purchased from Showa Denko K.K. (Japan). Polycaprolactone (PCL) was obtained from TRESNO (Poland). Chemicals used to prepare the culture media and cartridges for ion-exchange chromatography Bio-Scale Mini UNOsphere Q (strong anion exchanger) 5 mL and 1 mL, Bio-Scale Mini UNOsphere S (strong cation exchanger) 5 mL and 1 mL (GE Healthcare, Sweden) were purchased from Sigma-Aldrich (Germany). 0.5% biopolymer emulsions were prepared as previously described (Urbanek et al. 2021) and used in plate and turbidimetric assays to determine the biodegradation activity at each step of the purification procedure.

### Partial protein purification steps

After aerobic cultivation, the crude enzyme solution was obtained by centrifugation (10,000×*g* for 20 min, 4 °C) and paper filtration. The crude enzyme solution (350 mL) was initially concentrated with polyethylene glycol (PEG) to 158 mL. Next, ammonium sulfate (AS) was added to the solution in four steps to 20, 40, 60 and 80% saturation at 4 °C with stirring, respectively (Chua et al. 2013). The mixtures were centrifuged at 17,000×*g* at 4 °C for 20 min. Obtained pellets were dissolved in 20 mM Tris–HCl buffer (pH 8) and were treated as enzyme solutions marked as 20, 40, 60 and 80% AS, respectively. Subsequently, plate assays indicating biodegradation activity were performed. 80 µL of enzyme solutions were applied into wells in MM medium containing emulsified bioplastics. The halo zones formed during incubation corresponded to biodegradation activity of fractions. The enzyme solution with the highest activity was dialyzed, clarified and loaded onto Bio-Scale Mini UNOsphere Q 5 mL (GE Healthcare, Sweden) or Bio-Scale Mini UNOsphere S 5 mL (GE Healthcare, Sweden) cartridges using a Bio-Rad Duo Logic LP chromatography system (Bio-Rad, USA). The cartridges were previously equilibrated with 20 mM Tris–HCl buffer (pH 8.0) and high-degassed buffer according to the manufacturer’s instructions. Elution was performed first isocratically with the same buffer and then with a linear gradient from 0 to 1 M NaCl (0–100% buffer B). The flow rate was maintained at 1 mL/min. The active fractions were pooled and dialyzed overnight against 20 mM Tris–HCl buffer (pH 8.0). The enzyme solution was then applied to Bio-Scale Mini UNOsphere Q 5 mL (GE Healthcare, Sweden) equilibrated with 20 mM Tris–HCl buffer (pH 9.0). The column was washed with the same buffer, and the active fractions were eluted isocratically and with a 0–1 M NaCl linear gradient. The active fractions were pooled again, dialyzed against 20 mM Tris–HCl buffer (pH 9.0) and then loaded onto Bio-Scale Mini UNOsphere Q 1 mL (GE Healthcare, Sweden) equilibrated with 20 mM Tris–HCl buffer (pH 9.0). The active fractions were eluted isocratically. All fractions obtained at every purification step were analyzed for enzymatic activity in a plate assays (described in “Biodegradation activity” section). The protein concentration was determined according to Bradford (Bradford 1976) using BSA as standard. The molecular mass of the purified proteins was determined by SDS-PAGE using 12.5% polyacrylamide gel (Laemmli 1970). Unstained SDS-PAGE Broad Range Standard (Bio-Rad) was used as a molecular weight marker.

### Biodegradation activity

Wells were aseptically cut in solid MM medium supplemented with 0.1% bioplastic emulsions. 100 µL cell-free extracts or fractions collected during IEC were applied to each well. The plates were incubated for up to 24 h at 40 °C. After incubation, the emerging clean zones were observed as a result of enzymatic hydrolysis. The visible halo zone confirmed the presence of enzymes with biodegradation activity in the applied samples.

Moreover, the activity of the enzyme solutions was determined by measuring the decrease in turbidity of homogeneous PBSA, PBS and PCL suspensions according to García-Hidalgo et al. (2012) with slight modifications (García-Hidalgo et al. 2012). Briefly, a total of 3 mL of the standard reaction mixture containing 50 mM Tris–HCl buffer (pH 8.0), 2 mM CaCl_2_ and 200 μg/mL of sonicated homogeneous suspension of polyesters (1 mg/mL) in deionized water was pre-incubated at 40 °C for 5 min. Then, the reaction was started by adding the enzyme solution and the decrease in turbidity was measured every 5 min at 630 nm during the 40 min incubation period at 40 °C. Controls without enzymes were carried out in parallel to ascertain possible non-enzymatic hydrolysis of the polyesters. All activity assays were performed in duplicate. One unit (U) of enzyme activity was defined as the amount of enzyme which catalyzes a decrease of 0.01 absorbance units per minute at 630 nm under the assay conditions (García-Hidalgo et al. 2012). Protein concentration was determined as previously described (Bradford 1976) and the specific activity (U/mg protein) was calculated.

### Matrix-assisted laser desorption-time-of-flight (MALDI-TOF) analysis

Single bands obtained in SDS-PAGE were cut from the gel and analyzed by MALDI-TOF mass spectroscopy using single fragmentation (only one mass spectroscopy—MS—analysis) and double fragmentation of given peptides (MS/MS) in 4800 Proteomics Analyzer (AB SCIEX). The MASCOT database with similarity search against SwissProt database (https://www.expasy.org/resources/uniprotkb-swiss-prot) with a taxonomic restriction to fungi was used to identify proteins based on mass spectroscopy data (Wojtusik et al. 2018).

### Phylogenetic analyses

Subsequently, the obtained protein sequences were compared with known proteins with proved biodegradation activity within the BLASTp tool (Altschul et al. 1990). Multiple sequence alignment was built within ClustalOmega (https://www.ebi.ac.uk/Tools/msa/clustalo/) (Madeira et al. 2019) and next edited and visualised within Jalview (Waterhouse et al. 2009). The analysis was conducted to reveal any similarities of hydrGB10I and chitGB10I to the other known hydrolases involved in biodegradation such as depolymerases and cutinases. HydrGB10I was also compared to other members of the glycoside hydrolase family 81, and chitGB10I to chitinases. Protein sequences restricted to fungal proteins were retrieved from NCBI (National Centre for Biotechnology Information) (https://www.ncbi.nlm.nih.gov), UniProt (https://www.uniprot.org) and PMBD (Plastics Microbial Biodegradation Database) (http://pmbd.genome-mining.cn/home) databases.

Evolutionary phylogenetic analyses were performed within MEGA-X software using the neighbour-joining method (Kumar et al. 2018). Both the accession numbers and the names of the selected sequences are given under the alignment results presented in Additional file 1: Figs. S4, S5. The percentage identity of hydrGB10I and chitGB10I to other sequences is coloured blue. The most conserved residues in each group are the most intense in colour, and the least conserved are the palest.

PSSA (protein sequence-structure analysis) and homology modelling were performed within RaptorX (Källberg et al. 2012). The three-dimensional structures of the identified proteins were visualized by PyMOL (Schrödinger 2010). SWISS-MODEL (https://swissmodel.expasy.org), accessible via the Expasy webserver (Waterhouse et al. 2018) was also used to model protein structure homology modelling to obtain more precise information such as GMQE (Global Model Quality Estimate) value.

### Amino acid sequences

Accession numbers for amino acid sequences of proteins in GenBank are KFY49210.1 and KFY57494.1.

## Results

### Inoculum and enzyme production

Cold-adapted fungus *Geomyces* sp. B10I with the ability to degrade bioplastic (Additional file 1: Fig. S1) was cultivated aerobically in 2xYT medium supplemented with Tween 80. The addition of Tween 80 was necessary for the extracellular secretion of enzymes. It was observed that the biodegradation activity exhibited by the fungus was closely related to the release of spores (Additional file 1: Fig. S1). Thus, the culture was stopped when spore release was observed under the microscope. Cell-free broth containing the enzymes was obtained by centrifugation and filtration and was used as crude enzyme solution.

### Partial protein purification steps

**Fig. 1:**
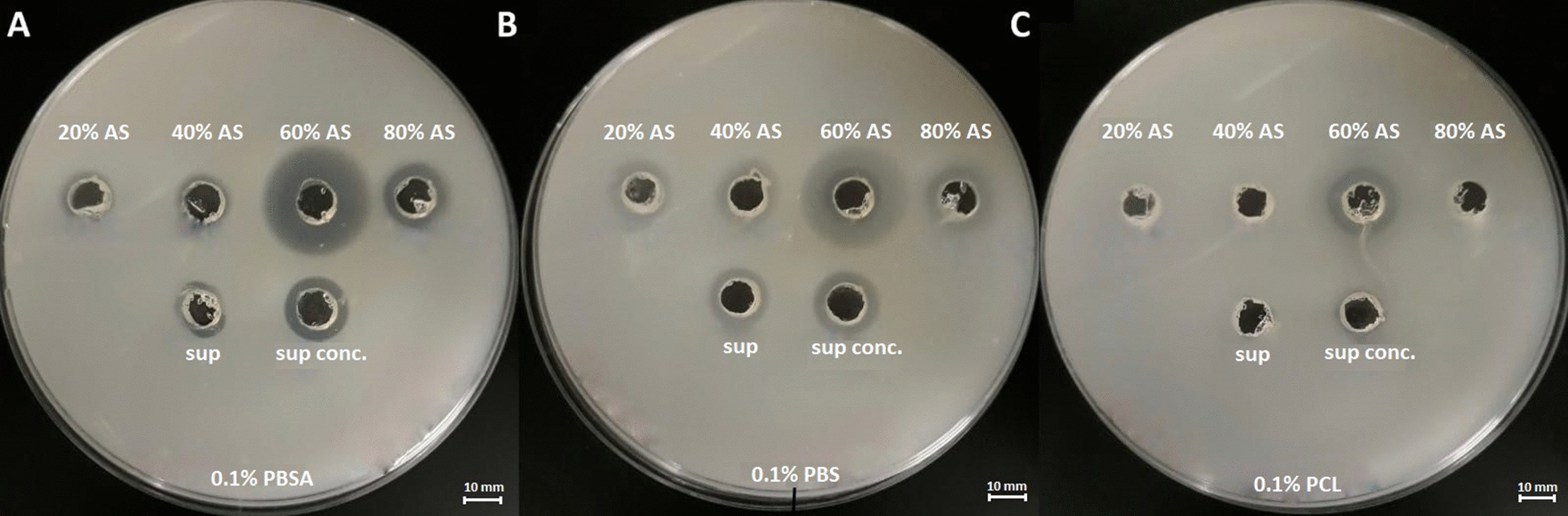
Comparison of enzymatic activity in a plate assay of cell-free broth (sup), concentrated cell-free broth (sup conc.) and fractions precipitated with (NH_4_)_2_SO_4_ (20, 40, 60 and 80% AS). The largest halo zone was observed in the fractions saturated with AS up to 60%. All fractions were applied on MM medium supplemented with **A** 0.1% PBSA; **B** 0.1% PBS; **C** 0.1% PCL

Three hundred and fifty millilitre of cell-free broth was initially concentrated to 158 mL. Next, the proteins were precipitated by the stepwise addition of ammonium sulfate (AS). Pellets obtained after centrifugation were dissolved in 20 mM Tris–HCl buffer and tested in a plate assays for hydrolytic activity. The largest halo zone was observed for 60% AS saturation (Fig. 1) and this fraction was selected for the purification process. The dialyzed and clarified fraction (11 mL) was loaded onto Bio-Scale Mini UNOsphere Q 5 mL, strong anion exchanger (GE Healthcare, Sweden). The enzyme with biodegradation activity was eluted both isocratically with 20 mM Tris–HCl buffer (pH 8.0) and with linear NaCl gradient (0–1.0 M) in the same buffer.

**Fig. 2:**
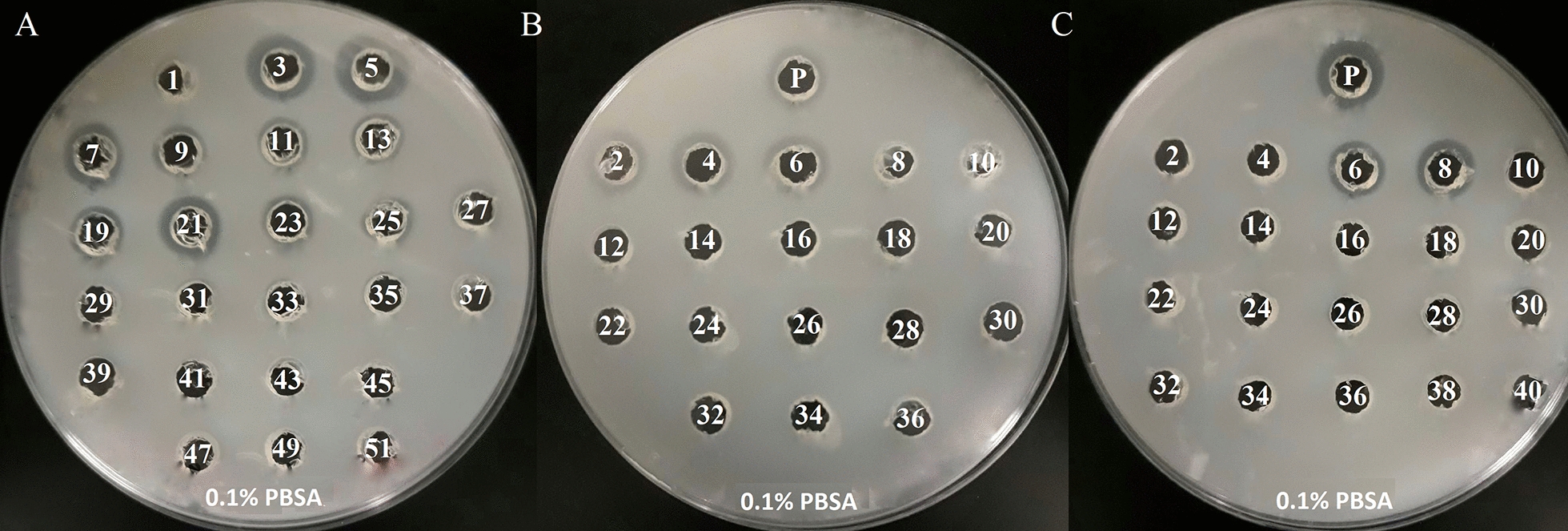
Biodegradation activity towards 0.1% PBSA in a plate assay exhibited by fractions collected throughout chromatographic steps: **A** pH 8.0, Bio-Scale Mini UNOsphere Q 5 mL; **B** pH 9.0, Bio-Scale Mini UNOsphere Q 5 mL; **C** pH 9.0, Bio-Scale Mini UNOsphere Q 1 mL. The numbers represent the consecutively collected factions; the letter P represents permeate

**Fig. 3:**
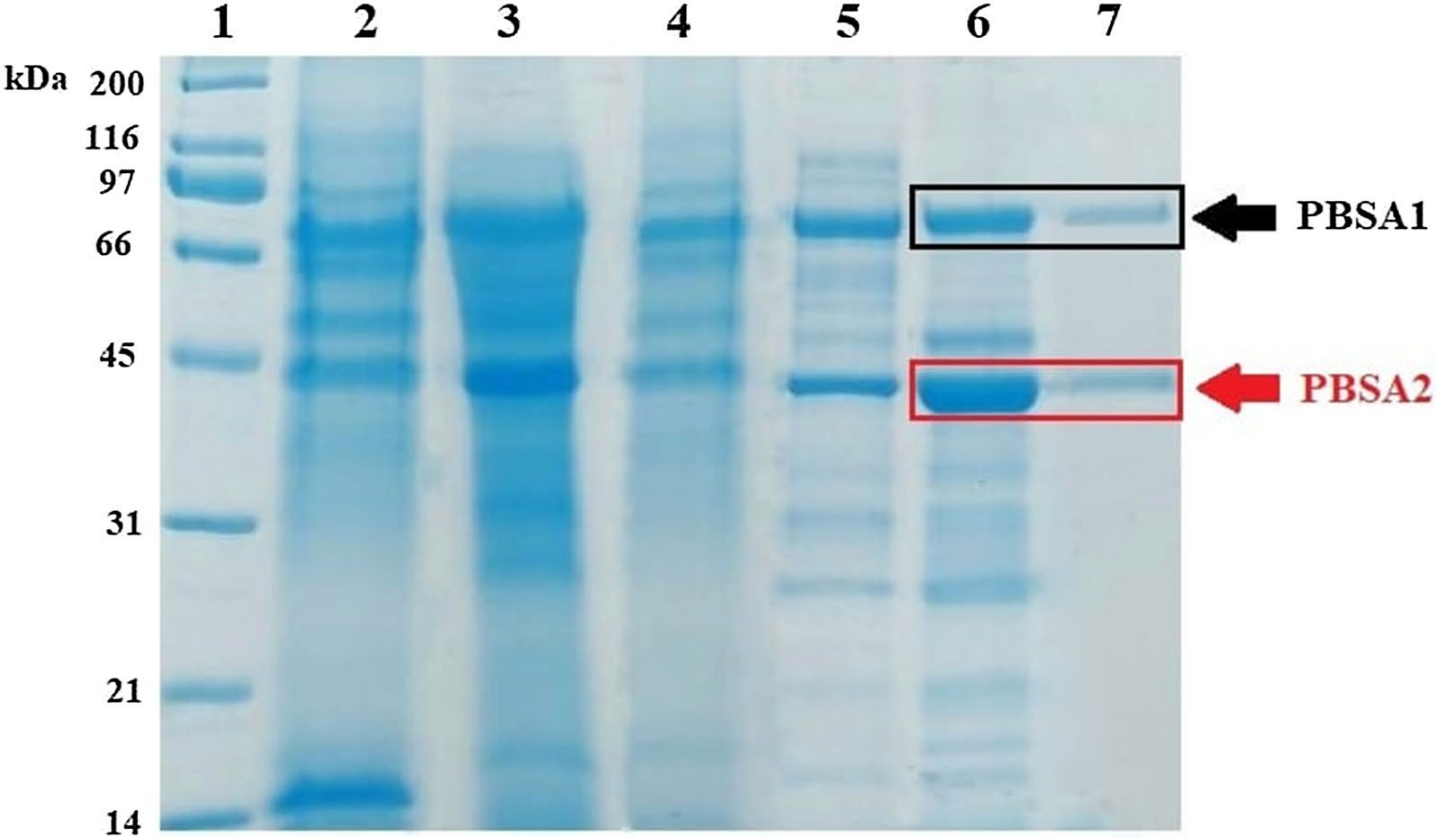
Electrophoretic profile of proteins. Lane 1—molecular weight standard (Unstained SDS-PAGE Broad Range Standard, Bio-Rad); lane 2—free-cell supernatant; lane 3—60% AS fraction; lane 4—pooled fractions (wash  +  gradient) exhibiting the activity after the first round of IEC; lane 5—pooled wash fractions after the second round of IEC; lane 6—pooled fractions after the third round of IEC; lane 7—fraction 6 after the third round of IEC showing only two bands and biodegradation activity. PBSA1—hydrGB10I, PBSA2—chitGB10I

The pooled, dialyzed and adjusted to pH 9.0 active fractions (Fig. 2A and Additional file 1: S2A) were applied onto the same column at pH 9.0. Here, the active enzymes were eluted isocratically (Fig. 2B and Additional file 1: S2B). Fractions were pooled again and applied onto Bio-Scale Mini UNOsphere Q 1 mL pH 9.0 and again eluted isocratically (Fig. 3C and Additional file 1: S3C). The peaks corresponding to the collected fractions with biodegradation activity in the plate assays are marked as red squares in the chromatograms (Additional file 1: Fig. S2). An application of active fraction onto cartridge packed with strong cation exchanger gave no result in purification (data not shown). During the protein purification steps, fractions containing enzymatically active proteins, i.e., cell-free broth, 60% AS fraction, pooled fraction from isocratic elution and fractions eluted in gradient were analysed by SDS-PAGE. Despite the fact, that fraction no. 6 collected after the third round of IEC did not bind to the resin, showed only two bands in SDS-PAGE (lane 6, Fig. 3) and exhibited hydrolytic activity in the plate assay towards PBSA (well 6, Fig. 2C). Similar effect was observed in a plate assays towards PBS and PCL (data not shown). This fraction contained 2.05 mg of partially purified proteins calculated by the Bradford measurement.

The specific biodegradation activity of the partially purified enzymes was measured by evaluating their ability to hydrolyze the PBSA emulsion in a turbidimetric assay. Although the activity of partially purified enzymes was confirmed by the plate assay, no reduction in the turbidity of homogeneous PBSA was observed. The yield and purification factors in the ammonium sulfate precipitation step were 31.31% and 14.16-fold, respectively, and the specific activity for PBSA was 91.89 U/mg (Table 1).

**Table 1:** Purification of native enzymes from the cell-free broth of *Geomyces* sp. B10I. The activity was measured in a turbidimetric assay with PBSA as a substrate for enzyme activity. Unsuccessfully, it was not possible to determine the activity of purified enzymes after the IEC procedure. The assays were performed in duplicate

	Volume (mL)	Protein (µg/mL)	Activity (U/mL)	Total protein (mg)	Total activity (U)	Specific activity (U/mg)	Purification factor	Yield (%)
Free-cell broth	350.0	77.31 ± 0.71	4.93 ± 0.01	27.06 ± 0.25	1.725.5 ± 4.95	63.77 ± 0.77	1	100
Concentrated free-cell broth	158.0	139.16 ± 2.56	10.76 ± 0.14	21.99 ± 0.4	1.700.08 ± 22.34	77.32 ± 0.40	1.21	98.52
60% (NH_4_)_2_SO_4_	11.0	52.47 ± 2.67	54.82 ± 2.8	0.66 ± 0.03	592.02 ± 15.24	903.22 ± 22.71	14.16	34.31

Next, the enzymatic activity towards different types of polyesters was compared using a turbidimetric assay. The results suggest that 40 °C is the optimal temperature for enzyme activity. The activity expressed in U/mL was twice as high as the activity in the other temperature variants for all polyesters. Moreover, the highest activity was indicated for PBS and the lowest for PCL (Table 2). It should be noted that a decrease in absorbance was also observed at 30 and 20 °C. Activities at 20 °C were half the value of the activity at 40 °C. Negative controls (buffer instead of enzyme mixture) indicated no decreases in turbidity.

**Table 2:** Biodegradation activity of free-cell broth after concentration and precipitation with (NH_4_)_2_SO_4_ towards polyesters: PBSA, PBS and PCL at different temperatures expressed in U/mL measured in turbidimetric assay. The experiment was performed in duplicate

	20 °C			30 °C			40 °C		
	PBSA	PBS	PCL	PBSA	PBS	PCL	PBSA	PBS	PCL
Concentrated free-cell broth	44.72 ± 0.34	80.96 ± 0.68	16.8 ± 0.23	40.48 ± 3.62	89.44 ± 2.04	8.64 ± 0.23	101.6 ± 5.09	124.44 ± 1.19	39.28 ± 4.41
60% (NH_4_)_2_SO_4_	165.6 ± 1.13	205.6 ± 0.57	60.88 ± 0.34	221.4 ± 3.62	309.4 ± 8.77	50.88 ± 1.58	369.6 ± 7.07	466.6 ± 11.60	65.76 ± 2.49

### Matrix-assisted laser desorption-time-of-flight (MALDI-TOF) analysis

**Fig. 4:**
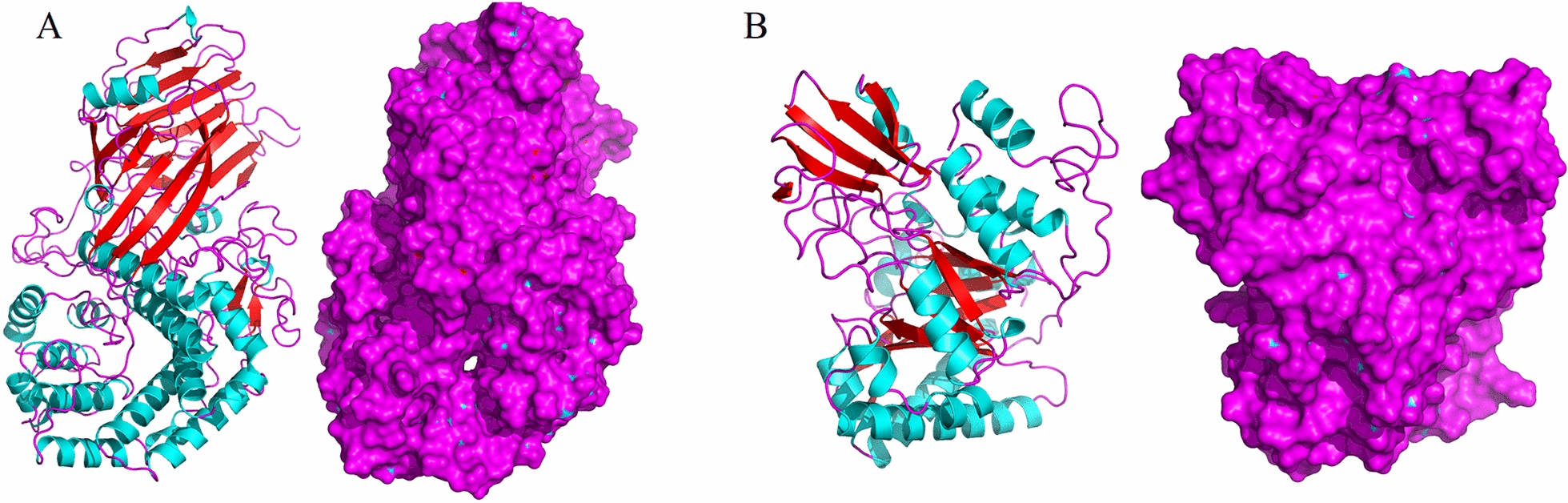
Model of **A** hydrGB10I (hydrolase) and **B** chitGB10I (chitinase) visualized within the PyMOL based on results of homology modelling obtained from RaptorX and COACH servers. Models present the three-dimensional structure of proteins. The secondary structure elements are coloured in blue (α-helices), red (β-sheets) and pink (loops). The surface structure is coloured in pink

The fractions collected during the purification process showing biodegradation activity in the plate assay were separated by SDS-PAGE. Flow-through fraction after the 3rd round of IEC showed activity (well 6) and revealed only two bands (Fig. 4, lane 6).

Bands named PBSA1 (hydrGB10I) and PBSA2 (chitGB10I) were separated from the gel (two repetitions of each band) and analyzed by MALDI-TOF. The results consisted of the combined peptide fingerprint analysis and peptide fragmentation of the bands. Additional file 1: Fig. S3 shows the peptide fingerprint result of partially purified proteins. Analysis revealed that hydrGB10I contained 714 aa with a predicted molecular mass of 77.248 kDa and a calculated pI 6.75, while chitGB10Icontained 428 aa with a molecular mass of 46.482 kDa and pI of 8.13. The obtained amino acid sequences (Additional file 1: Fig. S4) were compared with known sequences within the BLASTp tool. The search indicated that both hydrGB10Iand chitGB10I proteins showed identical values (100% identity, 0.0 e-value and 100% coverage) to the proteins characterized as hypothetical proteins from *Pseudogymnoascus* sp. VKM F-4515 with accession numbers KFY49210.1 and KFY57494.1, respectively. Both proteins contained conserved domains of glycoside hydrolases. The conserved domain of hydrGB10I belonged to the glycosyl hydrolase 81 superfamily. In turn, protein chitGB10I contained a domain of glycosyl hydrolase family 18 (GH18_chitinase). We found that 10 chitGB10I residues: Tyr47, Phe75, Asp171, Asp173, Glu175, Met241, Tyr243, Asp244, Tyr297, Trp382 appeared in the sequence of the glycosyl hydrolase 18 family as an active site. This active site was found in chitinase C from *Bacillus circulans*, chitinase B from *Saccharophagus degradans* 2–40 or chitinase from *Burkholderia dolosa* AU0158. The analysed proteins were named hydrGB10I and chitGB10I for PBSA1 and PBSA2 bands, respectively. The structure homology conducted within the Swiss-Model server (https://swissmodel.expasy.org/) suggested that both proteins are monomers. The modelling results showed that the sequence of hydrGB10I was 30.13% identical with 0.96 coverage of the template structure of the glycoside hydrolase family 81 endo-[beta]-1,3-glucanase, whereas the sequence of chitGB10I was 59.44% identical with 0.92 coverage to the crystal structure of a chitinase CrChi1 from the fungus *Clonostachys rosea*. The GMQE value for PBSA1 was 0.70 and for PBSA2 0.78, which represents high reliability in the target–template alignment. In addition, a total of 42 and 781 templates were found to match the target PBSA1 and PBSA2 sequences, respectively. The three-dimensional structure of proteins was visualized within the PyMOL program and was presented in Fig. 4.

**Fig. 5:**
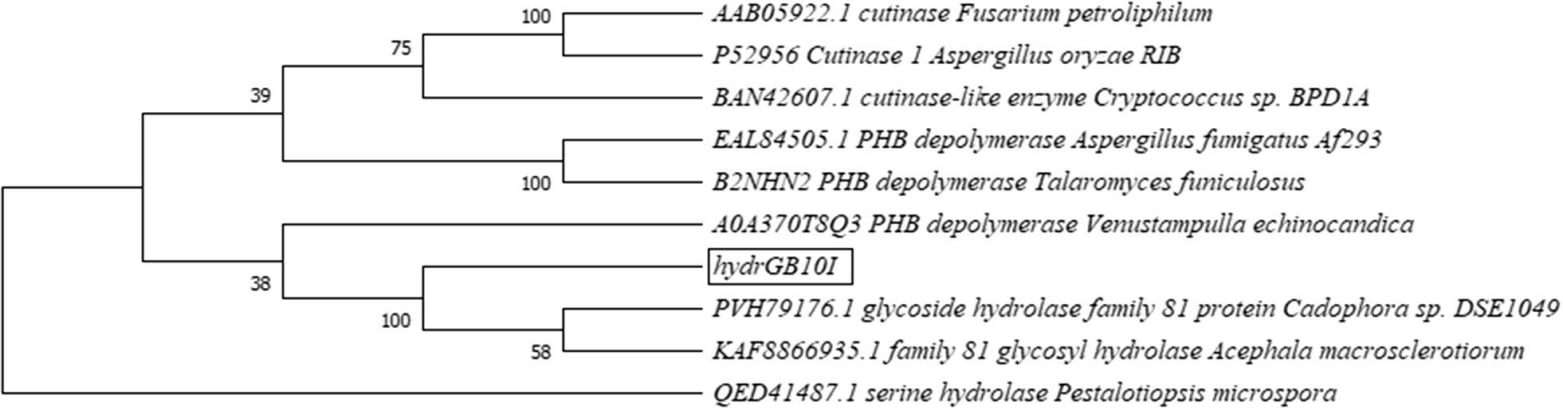
Neighbour-joining tree of hydrGB10I (*Geomyces* sp. B10I) and other enzymes with the biodegradation activity. The bootstrap consensus tree inferred from 1000 replicates and the percentage of replicate trees in which the associated taxa clustered together in the bootstrap test is shown next to the branches

**Fig. 6:**
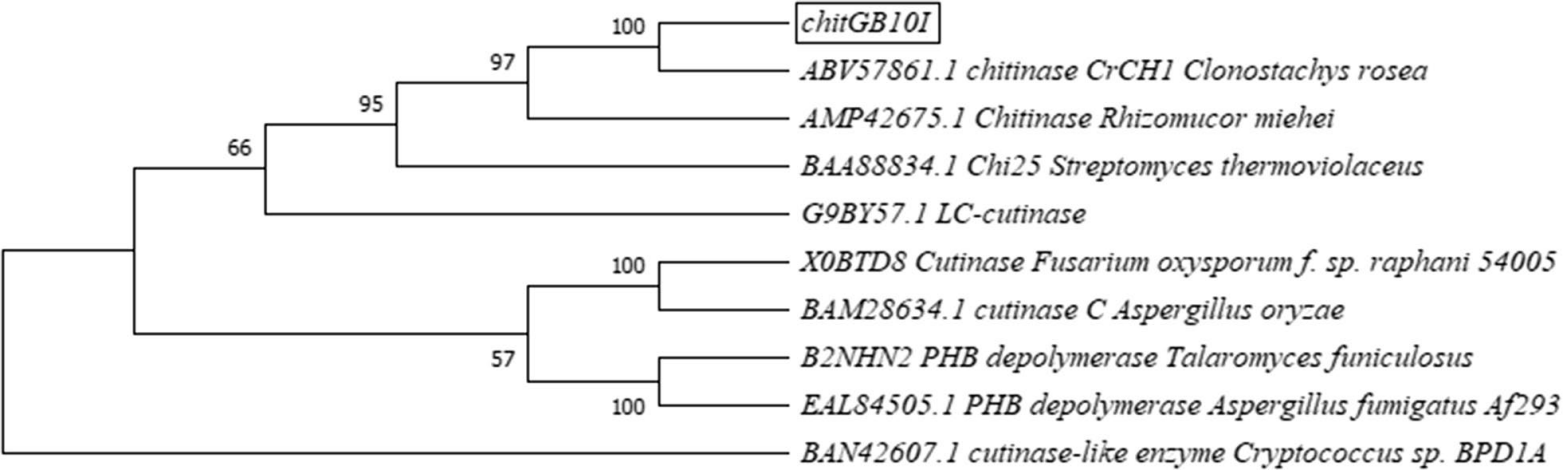
Neighbour-joining tree of chitGB10I (*Geomyces* sp. B10I) and other enzymes with the biodegradation activity. The bootstrap consensus tree inferred from 1000 replicates and the percentage of replicate trees in which the associated taxa clustered together in the bootstrap test is shown next to the branches

The analysis showed that the sequences have some conserved amino acids. The residues identified to be more than 60% conserved in the structure of hydrGB10I are Arg21, Pro100, Gln111, Asp129, Asn139, Pro148, Tyr151, Val192, Tyr208, Gly626, His628 and Gly667, whereas in chitGB10I sequence are Ala10, Ala12, Gly55, Ala61, Trp102, Gly169, Trp174, Leu197, Asn224, Trp249, Ala257, Gly293 and Gly333. The phylogenetic trees were conducted within the Mega-X software using the neighbour-joining method and are presented in Figs. 5,  6. We found that hydrGB10I is the most similar to glycoside hydrolase family (the bootstrap value  = 100) and distinct from other fungal enzymes with the biodegradation activity, whereas chitGB10I shows similarity to other chitinases and is the closest to LC-cutinase that exhibited the activity.

## Discussion

Extremophilic microorganisms are gaining more and more importance in biotechnological research and industrial applications. Psychrophiles have developed unique adaptive strategies to maintain their metabolic activity in the cold conditions which resulted in a stable membranes and a cell walls, unique compounds (e.g., exo-polysaccharides), proteins (the cold-shock protein, antifreeze/ice-nucleating protein) and genes (Arora and Panosyan 2019; Bhatia et al. 2021). Most importantly, they produce cold-active enzymes that catalyze biochemical reactions at low temperatures making them an attractive resource for biotechnological applications. Cold-active enzymes are primarily an alternative for the application of chemicals, lower the required temperature of a reaction and can prevent undesirable chemical reactions (Santiago et al. 2016). They are used in detergents, the paper and food industry or for bioremediation purposes (Arora and Panosyan 2019).

Cold-adapted fungus *Geomyces* sp. B10I has been reported to exhibit biodegradation activity against PBSA, PBS and PCL (Urbanek et al. 2021). In this study, we searched for enzymes that appear to be responsible for the biodegradation activity of *Geomyces* sp. B10I. As a result, two enzymes were partially purified, next identified and named hydrGB10I and chitGB10I. It should be emphasized that the complete purification of proteins may sometimes be complicated to perform. Problems usually arise from unknown properties and difficulties in selecting the appropriate resin binding conditions. The literature shows incomplete purification processes as in the case of PCL depolymerase from *Alcaligenes faecalis*. PCL depolymerase could not be purified to homogeneity due to inactivation during gel filtration. The enzyme did not bind to any other resin when IEC and HIC (hydrophobic interaction chromatography) was performed. Nonetheless, partial purification resulted in a 7.3-fold purity with a yield of 90.4% (Oda et al. 1997). In our study, partial purification resulted in 14.16-fold purity and SDS-PAGE analysis revealed the presence of two proteins in the active fraction collected during the IEC.

The amino acid sequences of both enzymes obtained from MALDI-TOF analysis were identical to hypothetical proteins of *Pseudogymnoascus* sp. VKM F-4515 (Leushkin et al. 2015). Up to date, this is the first report presenting the possible enzymatic function of these proteins and showing the cold-adapted fungus *Geomyces* sp. B10I (*Pseudogymnoascus* sp.) as a potential source of enzymes involved in the biodegradation of polyesters.

Both proteins contain conserved domains of glycoside hydrolases. Glycoside hydrolases (GHs) (EC 3.2.1.x.) are the key enzymes of carbohydrate metabolism. This extremely common group of enzymes hydrolyzes the glycosidic bond between carbohydrates or between the carbohydrate and non-carbohydrate moiety (Henrissat 1991). GHs are produced by a variety of microorganisms, including bacteria and fungi, where these enzymes are involved in cell wall recycling during carbon starvation, thereby generating energy and building blocks that can be used for maintenance and sporulation (van Munster et al. 2015). Furthermore, a conserved domain of the glycosyl hydrolase 81 superfamily (Ala15 through Gly713) was found in the hydrGB10I amino acid sequence. GH81 (EC 3.2.1.39) is a family of eukaryotic β-1,3-glucanases with endo-β-1,3-glucanase activity. Mouyna et al. (2016) proved that members of the GH81 and GH16 families play an important role in the morphogenesis of *Aspergillus fumigatus*. Endo β-1,3-glucanases are essential for the proper assembly of the conidial cell wall and thus for the efficient release of spores (Mouyna et al. 2016). In turn, analysis of the deduced amino acid sequence of chitGB10I revealed possession of the GH18 family catalytic domain (Asn44 through Asp387). The GH18 family, commonly known as chitinases (EC 3.2.1.14), is involved in carbohydrate metabolism along with chitin and polysaccharides degradation. Chitinases catalyze the random endohydrolysis of the 1,4-beta-linkages of N-acetylglucosamine in chitin and chitodextrins. Moreover, some chitinases can also hydrolyze related polymers. The size of chitinases varies from 20 kDa to about 90 kDa (Bhattacharya et al. 2007), which means that chitGB10I is of medium size (46.5 kDa). Fungal chitinases are not as well classified as bacterial chitinases and are identified by their similarity to a family 18 of bacteria and plants. It is supposed, that one of the roles of fungal chitinases is in the hyphal growth, branching and spore germination (Takaya et al. 1998; Sándor et al. 1998). For instance, multiple chitinase activity was observed in germinating spores in *Mucor rouxii* cells (Pedraza-Reyes and Lopez-Romero 1991). ChitGB10I, like other GH18 chitinases, contains a characteristic DxDxE amino acid sequence motif (^171^Asp-^172^Ile-^173^Asp-^174^Trp-^175^Glu), in which glutamate is a catalytic residue essential for activity (Malecki et al. 2020). As we observed the activity of *Geomyces* sp. B10I towards polyesters during sporulation, it is possible that both enzymes hydrGB10I and chitGB10I are responsible for the degradation abilities of this fungus.

Unfortunately, there is still little information available on the biochemical properties of fungal plastic-degrading enzymes or their structural characteristics compared to bacterial polyester-degrading enzymes. The obtained sequences of hydrGB10I and chitGB10I showed dissimilarities in comparison with other polyester-degrading fungal enzymes reported to date. The enzyme names, accession numbers, and fungal source used for the alignment are listed below in Figs. 5,  6 and below Additional file 1: Figs. S5 and S6. ChitGB10I was found to be similar to *Streptomyces thermoviolaceus* chitinase (Chua et al. 2013). To date, there is no more information on other chitinases showing biodegradation activity. In turn, hydrGB10I was more related to cutinases than to depolymerases, as is shown by the phylogenetic tree (Fig. 6).

Although the *Geomyces* sp. B10I is a cold-adapted fungus, the enzyme mixture tested in turbidimetric assays exhibited the highest biodegradation activity at 40 °C. This result shows that the optimal temperature for enzyme activity may differ from the growth temperature of the microorganism (Santiago et al. 2016). For instance, the optimal activity of pectinase from *Geomyces* sp. F09-T3-2 was indicated at 30 °C whereas the optimal temperature for fungal growth was 15 °C (Poveda et al. 2018). In turn, Mao et al. (2015) cultivated *Geomyces pannorum* at 20 °C while its *α*-amylase exhibited an optimal activity at 40 °C (Mao et al. 2015). The research of Engqvist (2018) suggests that the difference in growth temperatures and enzyme optima are caused by extrinsic factors such as increased action by chaperones, higher protein turnover rates, molecular crowding, or other undiscovered mechanisms (Engqvist 2018). However, taking into account the purpose of our research, it should be highlighted that the biodegradation activity was also detected at 20 and 30 °C. 50% of the activity at 40 °C was preserved at 20 °C, which is of great importance if the enzymes are to be used at ambient or room temperature. Thus the indicated biodegradation activity at 20 and 30 °C is a promising result in the search for enzymes adapted to lower temperatures than thermophilic. These enzymes could be an attractive targets for the biodegradation of bioplastic as well as other industrial applications. Cold-adapted enzymes have a few advantages over mesophilic orthologs in that they operate at low temperature, could reduce the energy cost in the reaction and attenuate side-reactions and they could be easily inactivated by heat (Nandanwar et al. 2020).

The information presented in this study is useful in the context of future research on the biodegradability of conventional plastics. The biodegradation activity of hydrGB10I and chitGB10I on fossil and aromatic plastics should be investigated. The production of these enzymes and their large-scale practical application should also be taken into account. Since *Geomyces* sp. is a fungus without GRAS status, it cannot be used directly as an enzyme producer. Moreover, purification of chitGB10I and hydrGB10I is a complicated process when enzymes are produced by the filamentous fungus. Therefore, research should focus on cloning sequences to other microorganisms for the independent secretion of enzymes and their easier purification.

## Supplementary Information

**Additional file 1: Figure S1.** *Geomyces *sp. B10I and its biodegradation activity. **A** Microscopic morphology at total magnification 25.2 ×: growing hyphae; **B** spore release; **C** biodegradation activity towards 0.1% PBSA in a plate assay; **D** Biodegradation activity of free-cell supernatant collected at different growth stages: red square represents free-cell supernatant obtained during hyphae growth, whereas black square from spore release stage; numbers represent the number of the flask. **Figure S2.** Chromatographic profiles of purification: **A** pH 8.0; **B** pH 9.0; **C** pH 9.0. The peaks with biodegradable activity are marked as a red square in the chromatograms. The flow rate was maintained at 1 ml/min for each purification step. **Figure S3.** Peptide fingerprinting MS result for bands signed as PBSA1 (**A**) and PBSA2 (**B**). Molecular weight of the analysed proteins was 77248 Da and 46482 Da, respectively. The search within BLAST revealed that both proteins had shown essentially identical properties (100% identity) to proteins characterized as a hypothetical proteins from *Pseudogymnoascus *sp. VKM F-4515 with accession numbers KFY49210.1 and KFY57494.1, respectively. **Figure S4.** Amino acid sequences of (**A**) hydrGB10I and (**B**) chitGB10I. **Figure S5.** Multiple sequence alignment of hydrGB10I with other enzymes exhibiting biodegradable activity. Conserved columns in each group are coloured in blue. Alignment conservation, quality, consensus and occupancy are displayed below the alignments. EAL84505.1—PHB depolymerase (*Aspergillus fumigatus *Af293); AAB05922.1—cutinase (*Fusarium petroliphilum*); BAN42607.1—cutinase-like enzyme (*Cryptococcus *sp. BPD1A); B2NHN2—PHB depolymerase (*Talaromyces funiculosus*); QED41487.1—serine hydrolase (*Pestalotiopsis microspore*); PVH79176.1—glycoside hydrolase family 81 protein (*Cadophora *sp. DSE1049); KAF8866935.1—family 81 glycosyl hydrolase (*Acephala macrosclerotiorum*); A0A370T8Q3—PHB depolymerase (*Venustampulla* *echinocandica*); P52956—Cutinase 1 (*Aspergillus oryzae *RIB40). **Figure S6.** Multiple sequence alignment of chitGB10I with other enzymes exhibiting biodegradable activity. Conserved columns in each group are coloured in blue. Alignment conservation, quality, consensus and occupancy are displayed below the alignments. BAA88834.1—Chi25 (*Streptomyces thermoviolaceus*); ABV57861.1—chitinase CrCH1 (*Clonostachys* *rosea*); AMP42675.1—chitinase (*Rhizomucor* *miehei*); X0BTD8—cutinase (*Fusarium oxysporum *f. sp. *raphani *54005); B2NHN2—PHB depolymerase (*Talaromyces funiculosus*); BAM28634.1—cutinase C (*Aspergillus oryzae*); BAN42607.1—cutinase-like enzyme (*Cryptococcus *sp. BPD1A), EAL84505.1—PHB depolymerase (*Aspergillus fumigatus *Af293); G9BY57.1—LC-cutinase. **Figure S7.** The scheme of purification procedure.

## Data Availability

The authors promise the availability of data and materials.

## Abbreviations

PCL: Poly(e-caprolactone)
PBS: Poly(butylene succinate)
PBSA: Poly(butylene succ inate-co-butylene adipate)
IEC: Ion exchange chromatography
MALDI-TOF: Matrix assisted laser desorption ionization-time of flight mass spectrometry
PHB: Polyhydroxybutyrate
PES: Polyethersulfone
PLA: Polylactic acid
PDLLA: Poly-DL-lactide
PLLA: Poly-L-lactide
BLAST: Basic local alignment search tool
MM: Minimal medium
PEG: Polyethylene glycol
BP: Biodegradable plastic
BSA: Bovine serum albumin
SDS-PAGE: Sodium dodecyl-sulfate polyacrylamide gel electrophoresis
NCBI: National Centre for Biotechnology Information
PSSA: Protein sequence-structure analysis
GMQE: Global model quality estimate
AS: Ammonium sulfate
HIC: Hydrophobic interaction chromatography
GH: Glycoside hydrolase
GRAS: Generally recognized as safe
