# Supplementary material for: Identification of novel extracellular putative chitinase and hydrolase from Geomyces sp. B10I with the biodegradation activity towards polyesters
Source: AMB Express. 2022 Feb 5;12:12. doi: 10.1186/s13568-022-01352-7 (PMC8818076; doi:10.1186/s13568-022-01352-7)
Supplement: Supplementary file 1 — Additional file 1: Figure S1. Geomyces sp. B10I and its biodegradation activity. A Microscopic morphology at total magnification 25.2 ×: growing hyphae; B spore release; C biodegradation activity towards 0.1% PBSA in a plate assay; D Biodegradation activity of free-cell supernatant collected at different growth stages: red square represents free-cell supernatant obtained during hyphae growth, whereas black square from spore release stage; numbers represent the number of the flask. Figure S2. Chromatographic profiles of purification: A pH 8.0; B pH 9.0; C pH 9.0. The peaks with biodegradable activity are marked as a red square in the chromatograms. The flow rate was maintained at 1 ml/min for each purification step. Figure S3. Peptide fingerprinting MS result for bands signed as PBSA1 (A) and PBSA2 (B). Molecular weight of the analysed proteins was 77248 Da and 46482 Da, respectively. The search within BLAST revealed that both proteins had shown essentially identical properties (100% identity) to proteins characterized as a hypothetical proteins from Pseudogymnoascus sp. VKM F-4515 with accession numbers KFY49210.1 and KFY57494.1, respectively. Figure S4. Amino acid sequences of (A) hydrGB10I and (B) chitGB10I. Figure S5. Multiple sequence alignment of hydrGB10I with other enzymes exhibiting biodegradable activity. Conserved columns in each group are coloured in blue. Alignment conservation, quality, consensus and occupancy are displayed below the alignments. EAL84505.1—PHB depolymerase (Aspergillus fumigatus Af293); AAB05922.1—cutinase (Fusarium petroliphilum); BAN42607.1—cutinase-like enzyme (Cryptococcus sp. BPD1A); B2NHN2—PHB depolymerase (Talaromyces funiculosus); QED41487.1—serine hydrolase (Pestalotiopsis microspore); PVH79176.1—glycoside hydrolase family 81 protein (Cadophora sp. DSE1049); KAF8866935.1—family 81 glycosyl hydrolase (Acephala macrosclerotiorum); A0A370T8Q3—PHB depolymerase (Venustampulla echinocandica); P52956—Cutinase 1 (Aspergillus oryzae RIB [file 13568_2022_1352_MOESM1_ESM.pdf]

Supplementary Materials:

Identification of novel extracellular putative chitinase and hydrolase from *Geomyces* sp. B10I with the biodegradation activity towards polyesters

Aneta K. Urbanek[1], Miguel Arroyo[2], Isabel de la Mata[2], Aleksandra M. Mirończuk[1,*]

[1] Department of Biotechnology and Food Microbiology, Faculty of Biotechnology and Food Science, Wroclaw University of Environmental and Life Sciences, Chełmońskiego 37, 51-630 Wrocław, Poland.

[2] Department of Biochemistry and Molecular Biology, Faculty of Biology, Universidad Complutense de Madrid, C. de José Antonio Novais, 12, 28040 Madrid, Spain.

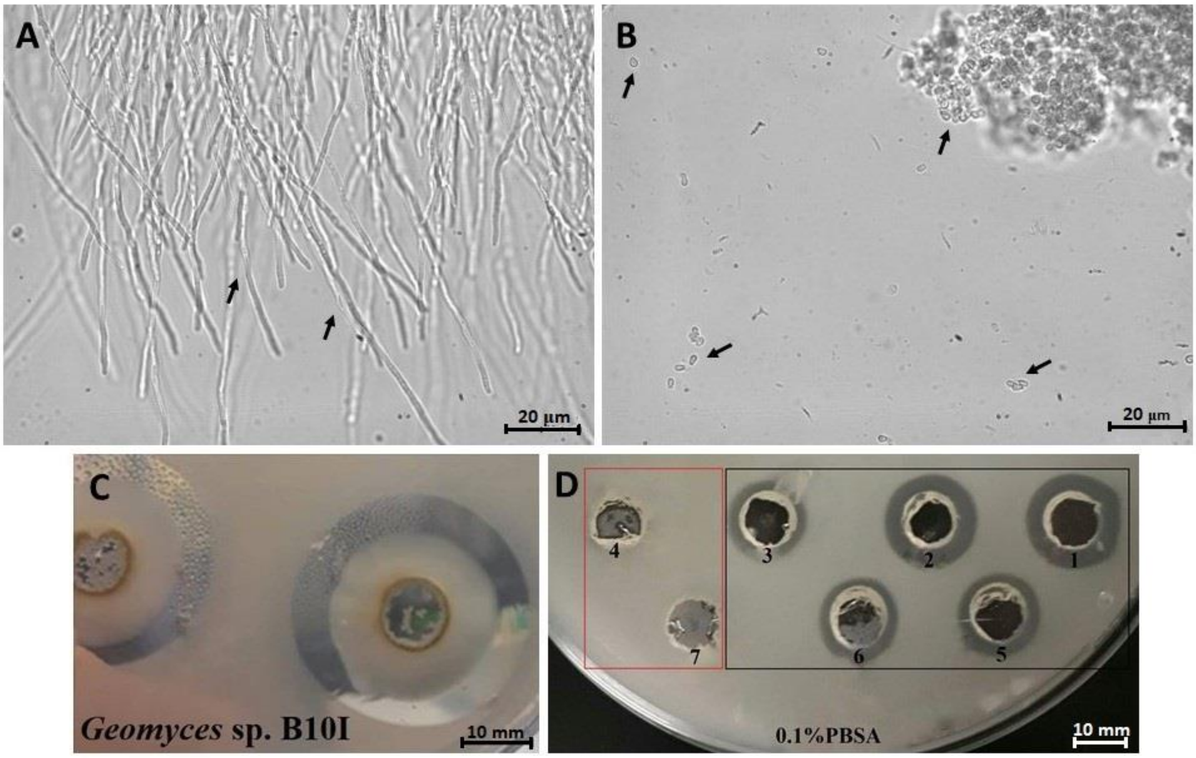

Figure S1. *Geomyces* sp. B10I and its biodegradation activity. (A) Microscopic morphology at total magnification 25.2 x: growing hyphae; (B) Spore release; (C) Biodegradation activity towards 0.1% PBSA in a plate assay; (D) Biodegradation activity of free-cell supernatant collected at different growth stages: red square represents free-cell supernatant obtained during hyphae growth, whereas black square from spore release stage; numbers represent the number of the flask.

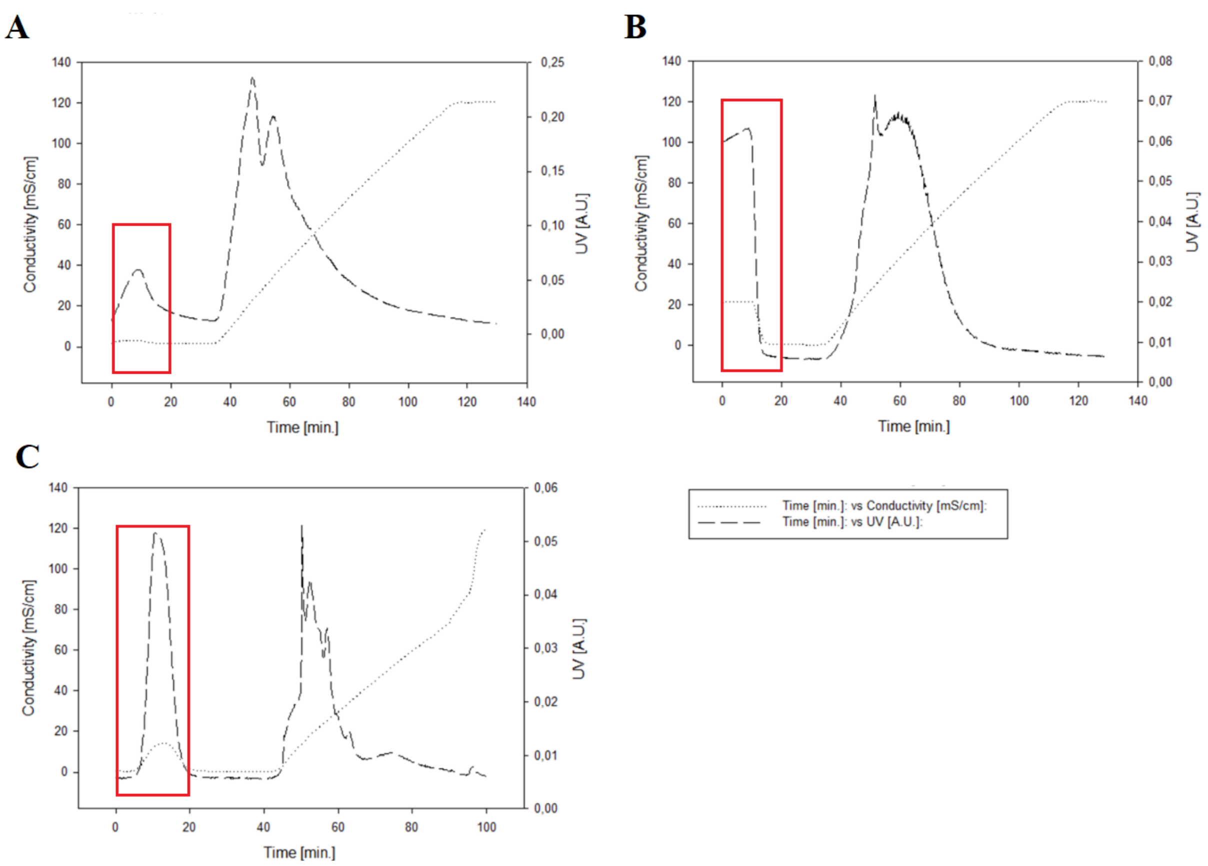

Figure S2. Chromatographic profiles of purification: (A) pH 8.0; (B) pH 9.0; (C) pH 9.0. The peaks with biodegradable activity are marked as a red square in the chromatograms. The flow rate was maintained at 1 mL/min for each purification step.

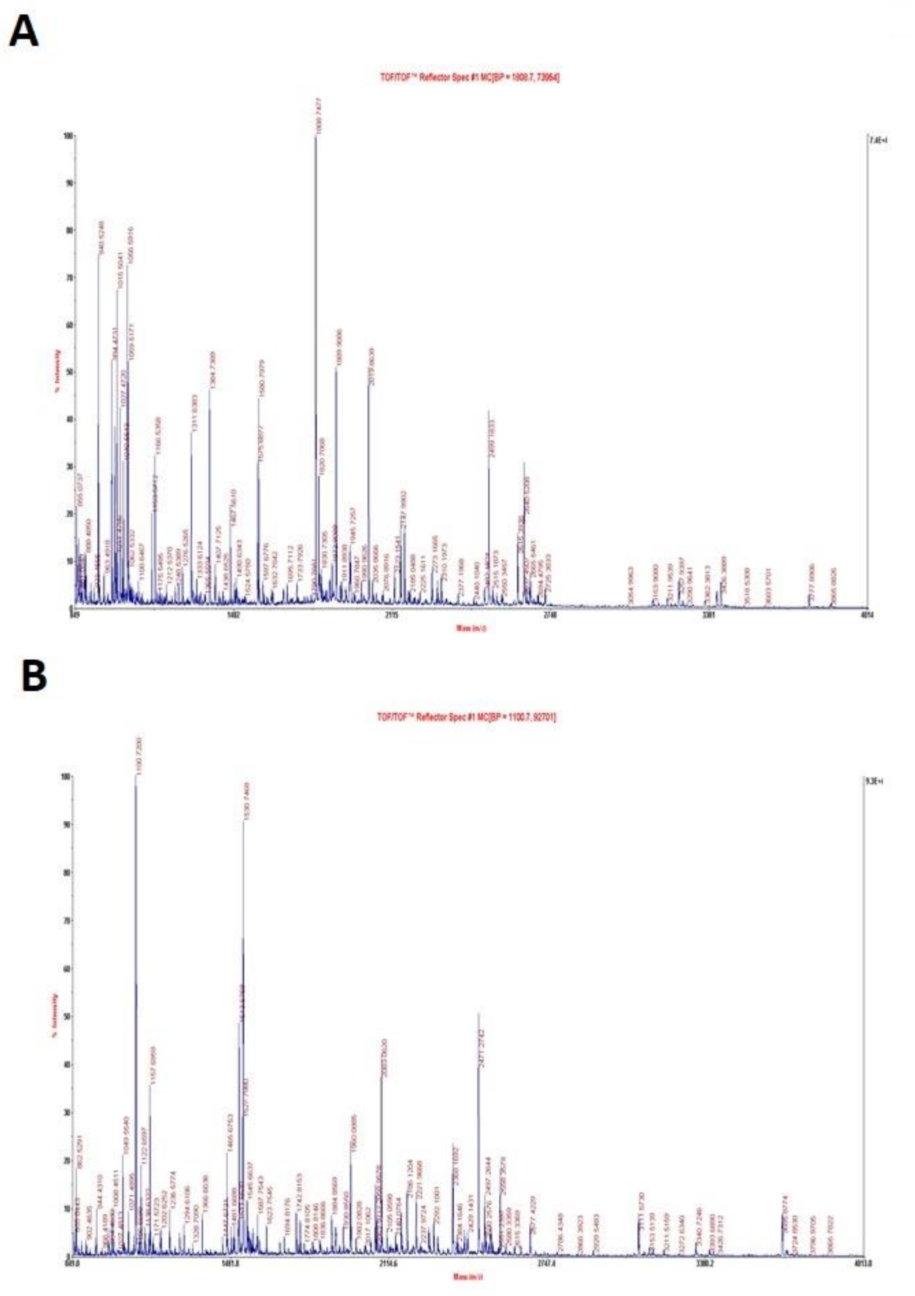

Figure S3. Peptide fingerprinting MS result for bands signed as PBSA1 (A) and PBSA2 (B). Molecular weight of the analysed proteins was 77248 Da and 46482 Da, respectively. The search within BLAST revealed that both proteins had shown essentially identical properties (100% identity) to proteins characterized as a hypothetical proteins from *Pseudogymnoascus* sp. VKM F-4515 with accession numbers KFY49210.1 and KFY57494.1, respectively.

A

MSSENIFQKALSKDAPPSMIASRPDHPVKRLGIAPQEGRPISTNKFYANFFLGGQNHATWTHPYSMLWSKGGGSSKS
WGLAVTHIEAKQQVFGPDPKASPAEYFVNPGGIQHVILSAAELEKSTVLTTDNLTSSSVNVNILASAGGKPAITFPLV
QGMGFITGIYNESTPILQSGVFFRSITQAKTAPKEGVIKYTIVLEDSSKWLVYAHSTSGQPLELSIANNSLIKATSKFQG
TLQIAKSPIDAAEAVYDAASGAYATGSALSGTAKGPAGTYTMTFSKGGLKDATLVMFALPHLVESFSSTTKAAATE
VKLQTTTKGVATGVVADSWTMEEKDMPVGMGFAPWSPSLGNIGTLSPSAISSIQKVAASDLKQDMMAQSNGDSFY
FSGKALAKFAQIIYASKDLVKDEALAKDGLTKLKAAFEVWTTNKNKYPLVYEKAWGGIVSTGGYVTGDSGTDFGN
TNYNDHHFHFAYFILAASYIGYLDPGWLASHKDYINTMVRDTANPSSEDPYFPVNRGFDWYNGHSWAKGLFESAD
GKDQESSAEDACFAYSLKMWGKTIGDANLEARGNLQLSVVARSISKYFLYTSDNTVQPKNFIGNKVSGILFENKIHH
TTYFGANIEYIQGIHMIPLLPSSSLTRTKKFVQEEWDTFFSDDRAKKVEGGWRGILFANLALIDPKQSYSFFSQESFDN
AWLDGGASRTWYMAQAAGLGGA

B

MTIGGSFKAAWAAVTGWRGKKTEGAAEGKADAAAAAPAAVAYKNAGYFVNWAIYGRNFQPAQLQAAQLTHVL
YAFANLRPDGSVFLSDTYADLEKHYPEDSWNEPGTNLFGCAKQIYLLKKKHRSMKVLLSIGGWTYSSNFAAAASTP
TTRALFVSTAVEIVKDLGFDGLDIDWEYPTNETEAKNYVLLLKACREGLDAYANANAKGYKFQLTIAAPAGPDKY
NILKMKEMDAYLDAWHLMAYDYAGSWSTVAGHDANLYPSKTVPEGTPYSTDKAVVDYIKAGVPAAKIIIGVPLY
GRSFQATEGMGKKFSGIGEGSWENGVWDYKVLPKAGATVKIDNDAKARYSYDPATKELISFDTVEDAKTKAEYVK
TKGLGGAMYWETSADRAGDKSLIGTFAGSFATLDKSQNLLSYPKSKYANMVAGMPS

Figure S4. Amino acid sequences of (A) hydrGB10I and (B) chitGB10I.

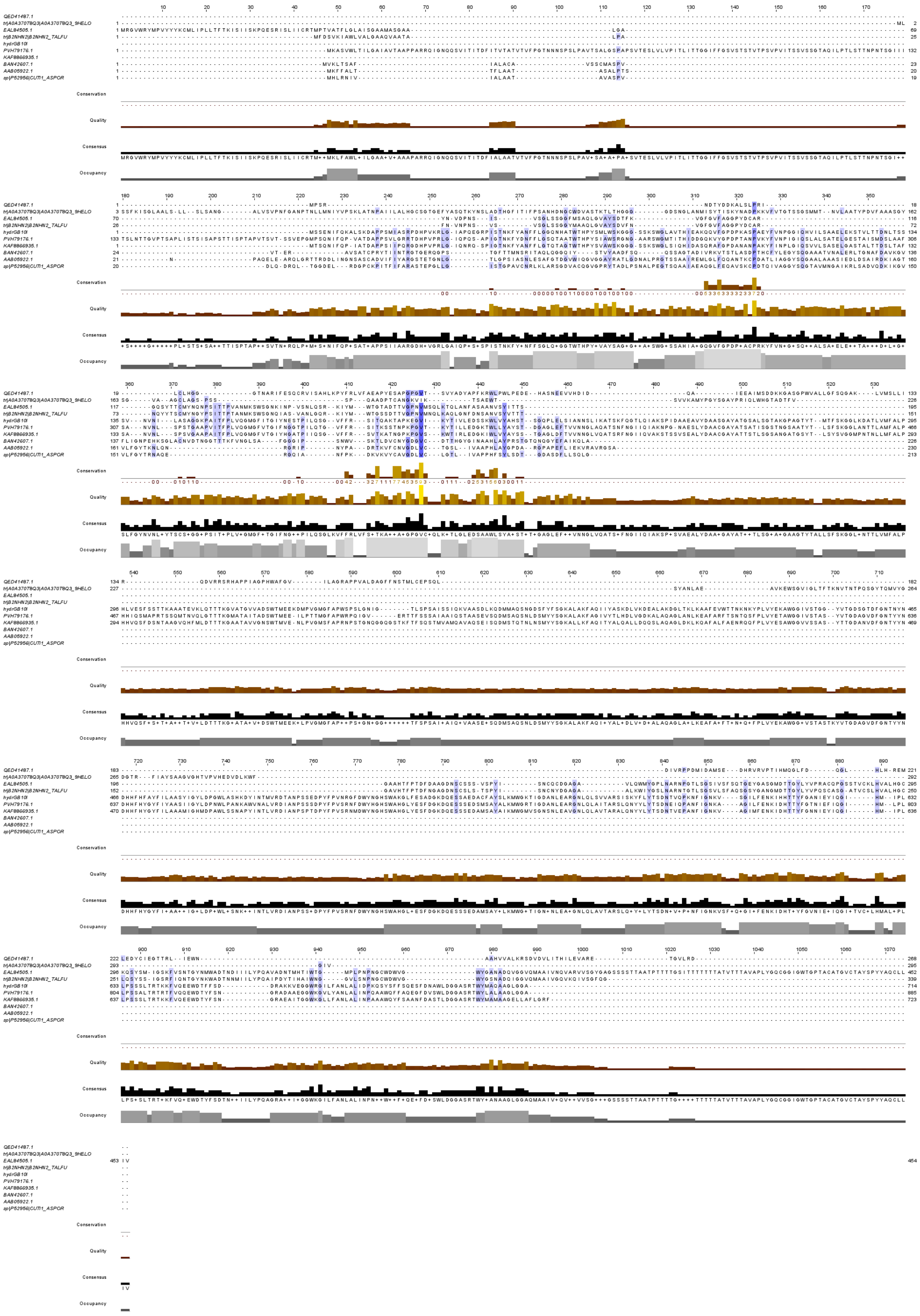
QED41487.1
tr|A0A370TBQ3|A0A370TBQ3_9HELO
EAL84505.1
tr|B2NHN2|B2NHN2_TALFU
hydrGB10I
PVH79176.1
KAF8866935.1
BAN42607.1
AAB05922.1
sp|P52956|CUTI1_ASPOR
Conservation
Quality
Consensus
Occupancy

Figure S5. Multiple sequence alignment of hydrGB10I with other enzymes exhibiting biodegradable activity. Conserved columns in each group are coloured in blue. Alignment conservation, quality, consensus and occupancy are displayed below the alignments. EAL84505.1 – PHB depolymerase (*Aspergillus fumigatus* Af293); AAB05922.1 – cutinase (*Fusarium petroliphilum*); BAN42607.1 – cutinase-like enzyme (*Cryptococcus* sp. BPD1A); B2NHN2 – PHB depolymerase (*Talaromyces funiculosus*); QED41487.1 – serine hydrolase (*Pestalotiopsis microspore*); PVH79176.1 – glycoside hydrolase family 81 protein (*Cadophora* sp. DSE1049); KAF8866935.1 – family 81 glycosyl hydrolase (*Acephala macrosclerotiorum*); A0A370T8Q3 – PHB depolymerase (Venustampulla echinocandica); P52956 – Cutinase 1 (*Aspergillus oryzae* RIB40)

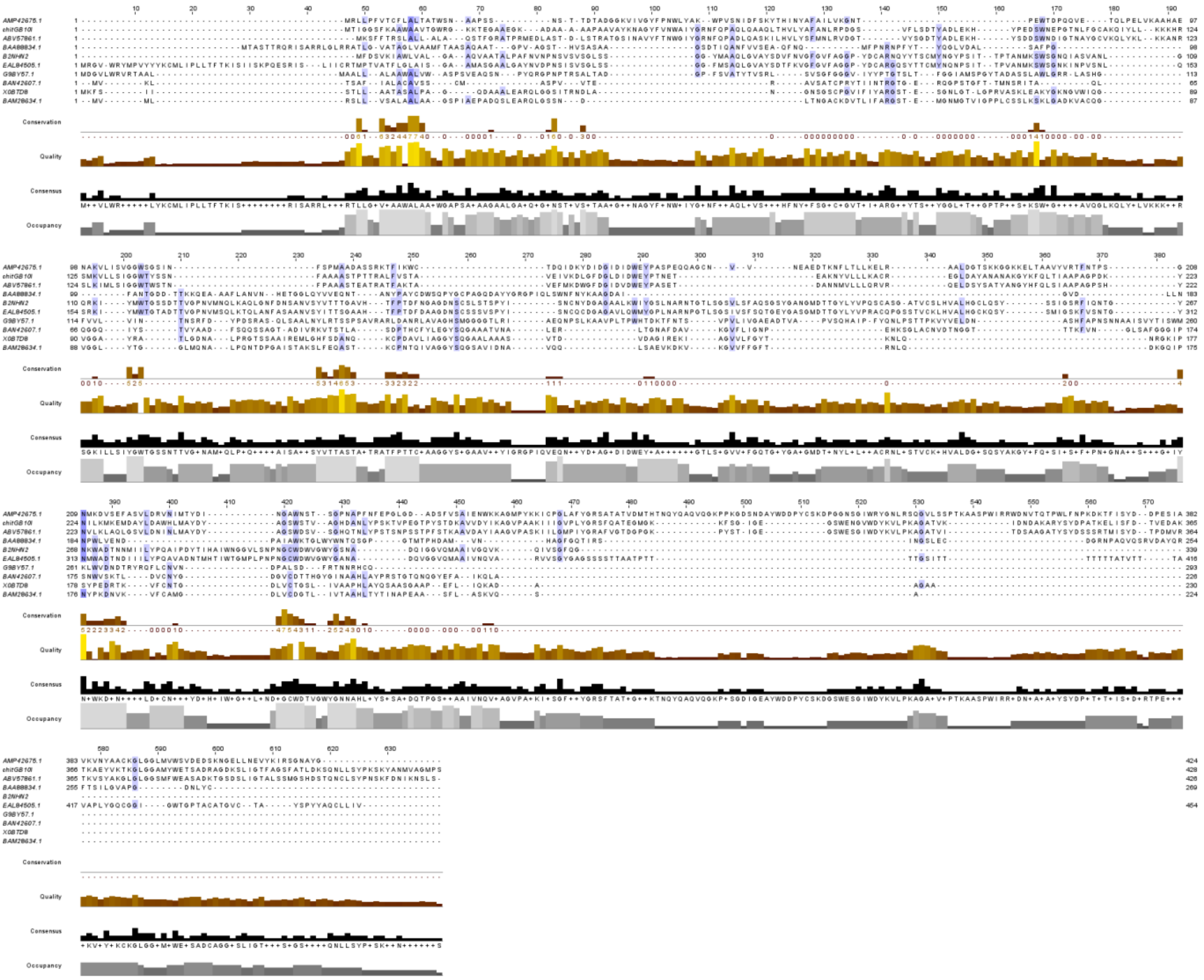

Figure S6. Multiple sequence alignment of chitGB10I with other enzymes exhibiting biodegradable activity. Conserved columns in each group are coloured in blue. Alignment conservation, quality, consensus and occupancy are displayed below the alignments. BAA88834.1 – Chi25 (*Streptomyces thermoviolaceus*); ABV57861.1 – chitinase CrCH1 (Clonostachys rosea); AMP42675.1 – chitinase (Rhizomucor miehei); X0BTD8 – cutinase (*Fusarium oxysporum f. sp. raphani* 54005); B2NHN2 – PHB depolymerase (*Talaromyces funiculosus*); BAM28634.1 – cutinase C (*Aspergillus oryzae*); BAN42607.1 – cutinase-like enzyme (*Cryptococcus* sp. BPD1A), EAL84505.1 – PHB depolymerase (*Aspergillus fumigatus* Af293); G9BY57.1 – LC-cutinase

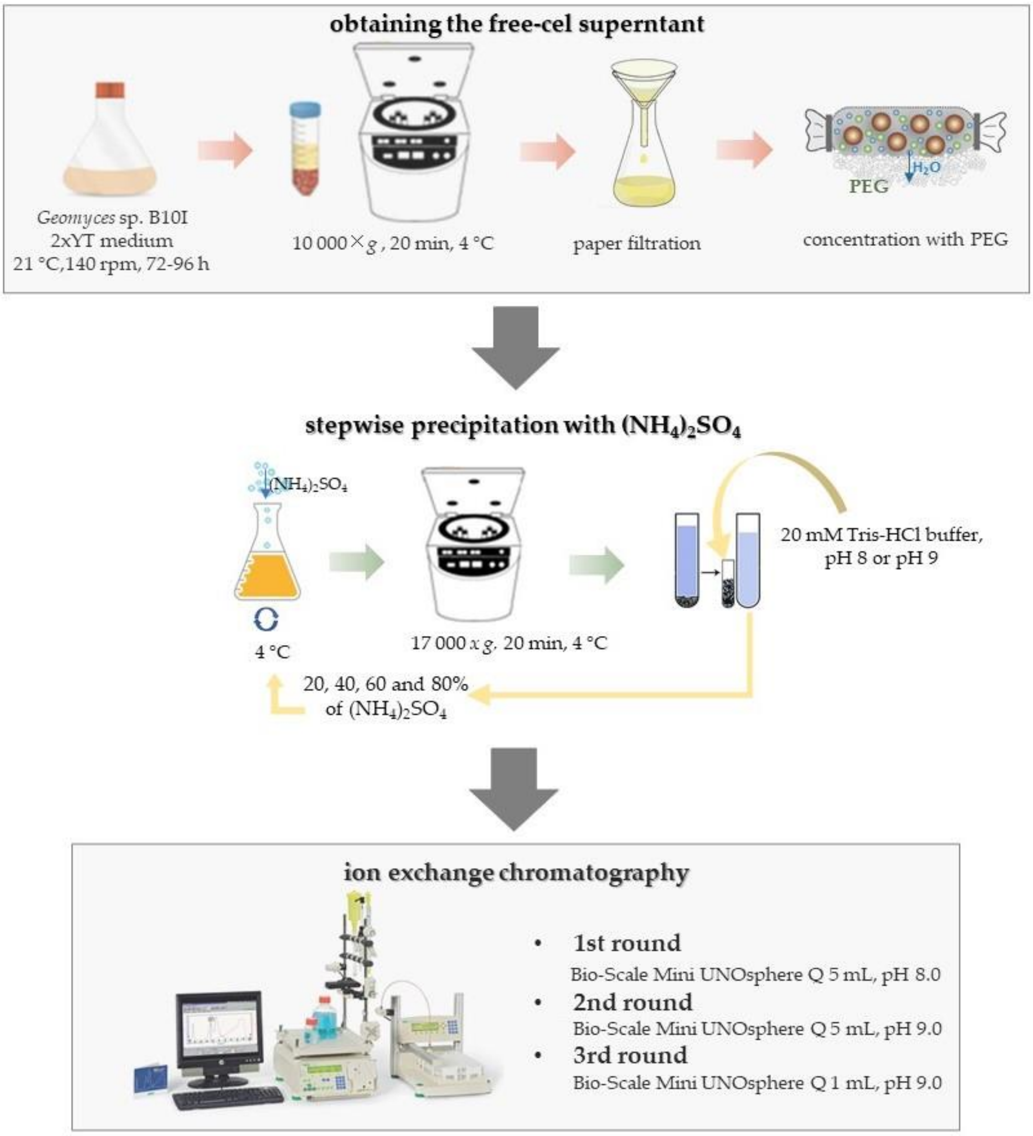

Figure S7. The scheme of purification procedure.
